# Clinical randomized trial study of hearing aids effectiveness in association with *Ginkgo biloba* extract (EGb 761) on tinnitus improvement^☆^

**DOI:** 10.1016/j.bjorl.2019.05.003

**Published:** 2019-06-18

**Authors:** Camila L. Radunz, Cristina E. Okuyama, Fátima C.A. Branco-Barreiro, Regina M.S. Pereira, Susana N. Diniz

**Affiliations:** a Universidade Anhanguera de São Paulo (UNIAN), Programa de Mestrado Profissional em Farmácia, São Paulo, SP, Brazil; b Universidade Federal de São Paulo (UNIFESP), Departamento de Fonoaudiologia, São Paulo, SP, Brazil; c Universidade Anhanguera de São Paulo (UNIAN), Programa de Mestrado Profissional em Farmácia e Mestrado e Doutorado em Biotecnologia e Inovação em Saúde, São Paulo, SP, Brazil

**Keywords:** *Ginkgo biloba*, Hearing aids, Hearing loss, Tinnitus, *Ginkgo biloba*, Prótese auditiva, Perda auditiva, Zumbido

## Abstract

**Introduction:**

Tinnitus is defined as the perception of sound without its actual presence in the environment. It has been the subject of a great number of studies, especially considering its consequences on patient's quality of life.

**Objective:**

This study aimed to investigate the effect of hearing aids and/or *Ginkgo biloba* extract EGb 761 on tinnitus in patients with hearing loss.

**Methods:**

This is a trial randomized-controlled treatment, parallel, double-blind, with three-arm. Thirty-three adults subjects were divided into three groups: group 1 — subjects undergoing drug therapy with *Ginkgo biloba* extract EGb 761; group 2 — individuals fitted with digital hearing aids; group 3 — individuals submitted to drug therapy with *Ginkgo biloba* extract EGb 761 and using hearing aids. The tinnitus handicap inventory and visual analogue scale were used to evaluate self-perception of tinnitus loudness and severity before treatment and 90 days after treatment.

**Results:**

This study demonstrated a significant correlation between tinnitus handicap inventory and visual analogue scale, before and after treatment. We observed a significant improvement in self-perception of tinnitus loudness and severity after 90 days of treatment with *Ginkgo biloba* extract EGb 761 and/or hearing aids. No correlation was found between tinnitus onset time and self-perception of tinnitus loudness and severity. Hearing aids were more effective in patients with a shorter tinnitus onset time and *Ginkgo biloba* extract was effective regardless of tinnitus duration.

**Conclusions:**

It was possible to prove the effectiveness of the hearing aids and/or *Ginkgo biloba* extract EGb 761 treatment, which shows success in the control of tinnitus contributing to the improvement of this symptom.

## Resumo

**Introdução:**

O zumbido é definido como a percepção de um som sem a sua presença real no ambiente e tem sido objeto de um grande número de estudos, especialmente devido às suas consequências na qualidade de vida do paciente.

**Objetivo:**

Investigar o efeito de próteses auditivas e / ou extrato de *Ginkgo biloba* EGb 761 sobre o zumbido em pacientes com perda auditiva.

**Método:**

Ensaio clínico randomizado controlado, paralelo, duplo-cego, com três braços. Trinta e três indivíduos adultos foram divididos em três grupos: Grupo 1- indivíduos submetidos à terapia medicamentosa com extrato de *Ginkgo biloba* EGb 761; Grupo 2- indivíduos equipados com próteses auditivas digitais; Grupo 3 - indivíduos submetidos à terapia medicamentosa com extrato de *Ginkgo biloba* EGb 761 e próteses auditivas. O *Tinnitus handicap inventory* e a escala visual analógica foram usados para avaliar a autopercepção de *intensidade* e da gravidade do zumbido antes do tratamento e 90 dias após o tratamento.

**Resultados:**

Este estudo demonstrou uma correlação significante entre o *Tinnitus handicap inventory* e a escala visual analógica, antes e após o tratamento. Observou-se melhoria significativa na autopercepção de *loudness* e da intensidade do zumbido após 90 dias de tratamento com extrato de *Ginkgo biloba* EGb 761 e/ou prótese auditiva. Não foi encontrada correlação entre o tempo de início do zumbido e a autopercepção da intensidade e gravidade do zumbido. As próteses auditivas foram mais eficazes em pacientes com menor tempo de início de zumbido e o extrato de *Ginkgo biloba* foi eficaz, independentemente da duração do zumbido.

**Conclusões:**

Foi possível comprovar a eficácia do tratamento com a prótese auditiva e/ou extrato *de Ginkgo biloba* EGb 761, o que demonstra sucesso no controle do zumbido e contribui para a melhoria desse sintoma.

## Introduction

Tinnitus has been the subject of a great number of studies, especially due to its consequences on patient's quality of life. It is defined as the perception of sound without its actual presence in the environment^1^ and is considered a symptom, not an ailment in itself, of anomalies from cochlear function and signal processing in the central nervous system.^2^ Commonly, tinnitus is associated with other diseases, and it is difficult to isolate the causes: otological ailments; cardiovascular alterations; metabolic, neurological and psychiatric diseases; odontological factors and possibly consequence of drugs, caffeine, nicotine and alcohol ingestion.^2^ Health professionals are increasingly involved in obtaining a more precise and accurate evaluation of patients, looking in addition for efficient and lasting therapeutic alternatives.^3^

Most patients with tinnitus (85–96%) have some degree of hearing loss,^4^ which shows that regardless of the hearing loss degree tinnitus has a greater impact on people's quality of life.^5^, ^6^ The positive correlation between hearing loss and tinnitus can be justified if one considers that the former may be the triggering factor of the latter, since damage or degeneration of the inner ear and of the vestibulocochlear nerve may cause this symptom.^5^ A conventional amplification Hearing Aid (HA) is one of the most frequent devices used to rehabilitate hearing loss.^6^ The HA has also been employed in the treatment of tinnitus, because it is an efficient alternative to minimize its perception by the patients and to initiate and facilitate the process of getting used to tinnitus.^7^

Drug therapy is used to treat tinnitus and several drugs/active principles are commonly used to minimize this symptom; among them we found lidocaine hydrochloride, carbamazepine, gabapentine, fluoxetine hydrochloride, sertraline hydrochloride, clonazepam, alprazolam, betahistine, cinnarizine, flunarizine and the *Ginkgo biloba* extract.^3^ Moreover, associations of different drugs used in the treatment of tinnitus with *G. biloba* extract^8^, ^9^ are also described in the literature, although these studies are controversial.

The extract from *G. biloba* leaves is rich in flavones and glycosylated flavonoids, terpenes, lactones and other constituents, which are responsible for the pharmacological properties of the extract.^10^ The extract is used in medicine and is prescribed for cognitive dysfunctions, including dementia^11^ and cerebrovascular insufficiency,^12^ recent memory loss, headache, vertigo^13^, ^14^ and tinnitus in addition to emotional instability accompanied by anxiety.^15^ The preparations containing *G. biloba* increase blood flow, with the resulting improvement of oxygen supply to the cells, protect tissues from the damage resulting from lack of oxygen. Furthermore, an inhibition of platelet aggregation has been found, thus being recommended in the case of deficit in the cerebral blood flow, which may give rise to memory loss, dizziness, headaches and tinnitus.^13^, ^14^ Studies have shown that terpene lactones such as ginkgolide B and bilobalide are the constituents of the extract responsible for these properties.^10^

Thus, in view of the therapeutic properties of Gingko and employment of HA in auditory rehabilitation, we determined to investigate the effect of the use of *G. biloba* extract EGb 761 and/or hearing aids (HA) on tinnitus in patients with hearing loss.

## Methods

### Study design

This is a trial utilizing randomized-controlled treatment, parallel, double-blind, with three-arm, (TRIAL: RBR-2SN8XV) registry at *Registro Brasileiro de Ensaios Clínicos* (ReBEC) (http://www.ensaiosclinicos.gov.br/). The random allocation process has consisted in generating a random sequence using an Excel file for randomization and used the random allocation rule, which chooses at random one of possible balanced assignments of the given number of subjects per treatment.

The examiners responsible for applying the questionnaires during the study were blinded to the intervention. In addition, an employee outside the research team inserted data into the computer in separate datasheets so that the researchers can analyze data without having access to information about the allocation.

### Participants

The participants’ selection process was performed after consultation with the otolaryngologist, who identified the individuals appropriately, generated the random allocation sequence, and enrolled and designated the participants for the interventions. The sample size (*n* = 35) was determined by the number of individuals submitted to medical consultation between July and August 2015 at an audiological center in Rondonópolis (MT), Brazil.

### Inclusion and exclusion criteria

Patients were informed about the study and invited to participate. All were completely free to refuse to participate without any loss to the treatment in the clinic. The inclusion criteria of the patients were: individuals over 18 years of age; complaint of tinnitus (uni- or bilateral) for at least 3 months; (single or bilateral) sensorineural or mixed hearing loss independent of degree and configuration. Both male and female were recruited. The exclusion criteria of the patients were: having used aspirin or acetylsalicylic acid (ASA) in the last month; having used of *G. biloba* in the last 3 months; on antidepressant use; diagnosed with compromised middle ear (otitis or tubal dysfunction) at the time of evaluation.

### Interventions

The groups were divided into: group 1 — patients (*n* = 11) who received drug therapy with *G. biloba* extract EGb 761, dose of 240 mg/day/patient; group 2 — patients (*n* = 11) who were equipped with Beltone® individual HA digital; group 3 — was composed of individuals (*n* = 11) who underwent both drug therapy with *G. biloba*, identical to Group 1, and to the HA. After starting the study, the treatment was performed for 90 days.

EGb 761 Equitam® leaf extract (patented/trademarked by Eurofarma Laboratórios S.A.), is a well-characterized complex mixture of biologically active constituents obtained from *G. biloba* leaves according to a standardized method.^16^ EGb 761 contains the active compounds: 24% of flavonoid glycosides, 6% of terpenoid trilactones, 5–10% of organic acids.^17^, ^18^, ^19^ The flavonoids and terpenoids are proposed as the biological active compounds of EGb 761 with pharmacological activity and different properties.^20^ The dose of 240 mg of EGb 761, supplied by Momenta, was administered daily by the oral route. Digital Beltone® HA composed of three basic components: a microphone, an amplifier and a speaker and obtained at a private Hearing Aids center (MT, Brazil). The route of administration of *G. biloba* should be mentioned, along with the oral doses.

### Clinical procedures

Participants were seen 5 times over a 6 month period for this longitudinal study. The study was performed in a clinical outpatient clinic of the audiological center. The services were provided by otorhinolaryngologists, speech therapists and researchers involved with the present study. The visits of the participants were divided into the following stages:

Visit 1: All the patients underwent an evaluation with otolaryngologists who guided and gave all the clarification regarding the research in question. Subsequently, the otolaryngologists referred these patients for adaptation of individual hearing aid, treatment with *G. biloba* or association of both.

Visit 2: During the hearing aids fitting, tinnitus counseling and education was provided by an audiologist and speech therapist. Information about *G. biloba* treatment and the questionnaires were presented to the subjects by researchers.

Visits 3 and 4: The questionnaires were applied to collect the description and evaluation of tinnitus from patients at a private audiological center. All participants answered the Tinnitus Handicap Inventory (THI), adapted by Brazilian Portuguese by Ferreira et al.,^21^ the only instrument translated and adapted for this language to investigate the severity of tinnitus and the visual analogue scale (VAS). Both THI and VAS were applied at the early (pre-treatment, Visit 3) stage and following 90 days of treatments (Visit 4).

Visit 5: After completing the research, all participants were individually called for clarification of the results obtained in the study by the researchers, accompanied by speech therapists.

### Outcome measures

This study compared the effect of the use of the individual hearing aid, the use of *G. biloba* preparation and the association between the hearing aid and the *G. biloba* extract, through the application of two questionnaires, THI and VAS, seeking an effective alternative to the treatment of tinnitus. As a secondary outcome mean percentage of variation of the THI score before and after treatment in relation to the tinnitus onset time was also analyzed.

#### THI

The THI is comprised of 25 questions, and the possible answers are: Yes, Sometimes or No. The scores given to the answers are, respectively: four, two and zero points. The total score varies from 0 to 100, and the higher the score the greater the effect of tinnitus on the patient's quality of life. This questionnaire was translated and culturally adapted to be applied to the population, and has proven to be a reliable tool to evaluate the damage caused by tinnitus to people's quality of life.^21^

#### VAS

The application of the VAS to patients with tinnitus consists in attributing a score from 0 to 10 for discomfort and another score for the intensity of the symptom, with the help of an appropriate rule,^22^ the first attribution being used in the questionnaire posed to the patient in this survey.

### Ethical approval

A favorable ethical opinion was granted from the Ethics Committee, under decision nº 1,062,277 (http://plataformabrasil.saude.gov.br/login.jsf), as it conforms to Resolution 466/2012, where all subjects of this study receive a treatment.

### Statistical analysis

The quantitative data and significant differences (*p < 0.05) were evaluated through ANOVA in the pre-treatment and post-treatment period for comparison between the groups (G1, G2 and G3). The relation between the tools used (THI and VAS) in the periods both before and after treatment was also analyzed in accordance with Pearson's correlation coefficient (*r*). Lastly, the relation between the tinnitus score measured by the different scales and the time of tinnitus (measured in months) was evaluated. The mean time (59 months) of tinnitus was calculated among all patients included in the study. The GraphPadPrism® 5 software was used for the biostatistical analyses.

## Results

**Figure 1:**
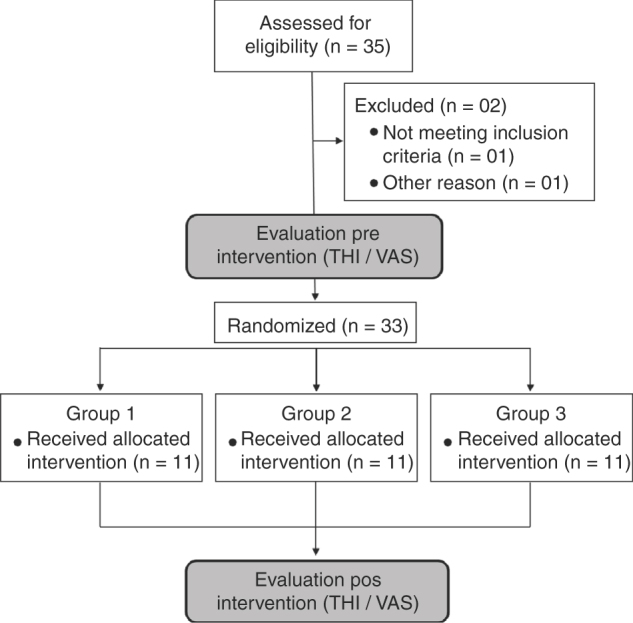
Flow diagram illustrating the participants number who were randomly assigned for each group.

From the population sample of 35 individuals recruited from July to August, 2015, two were excluded and 33 were allocated in 3 different groups as illustrated in Fig. 1. In addition, one individual with hearing loss and tinnitus with null score of VAS and THI was excluded from the study for this reason. Another patient was excluded because he did not present hearing loss but presented a severe tinnitus. Demographic information of participants of the study is shown in Table 1.

**Table 1:** Demographic summary data of participants by number of individuals, gender, age and time of tinnitus in months.

Variable	Participants (useable data)	Full population
Number of individuals (male/female)	33 (45.5%/54.5%)	35 (45.7%/54.3%)
Mean age in years (SD)	56.3 (16.8)	56.3 (16.8)
Mean time of tinnitus in months before treatment (SD)	58.9 (17.7)	55.9 (21.1)

SD, standard deviation.

**Figure 2:**
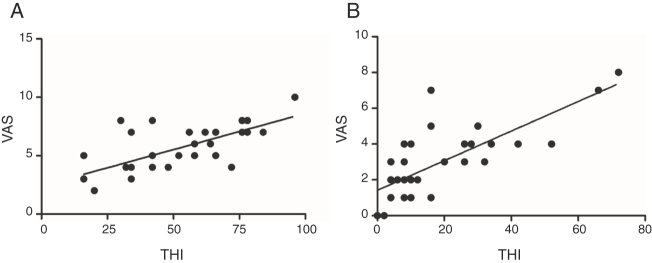
Pearson’s correlation of the scores obtained by the THI and VAS methodological instruments in the pre-treatment (A) of all the research volunteers (*r* = 0.6785; *p* < 0.0001) and in the posttreatment (B) of all volunteers; (*r* = 0.7519, *p* < 0.0001).

This study evaluated the correlation between the THI and VAS scales in the periods before and after treatment (Fig. 2). In accordance with Pearson’s correlation coefficient (*r*), we can observe the existence of a significant correlation (*r* = 0.6785; *p* < 0.0001) before the treatment (Fig. 2A). In the post-treatment period (Fig. 2B), a statistical dependency was also found, with a significant correlation between both tools (*r* = 0.7519; *p* < 0.0001). This means that the greater the value of VAS found, the greater is the expected value of THI (positive correlation), as shown in Fig. 2.

**Figure 3:**
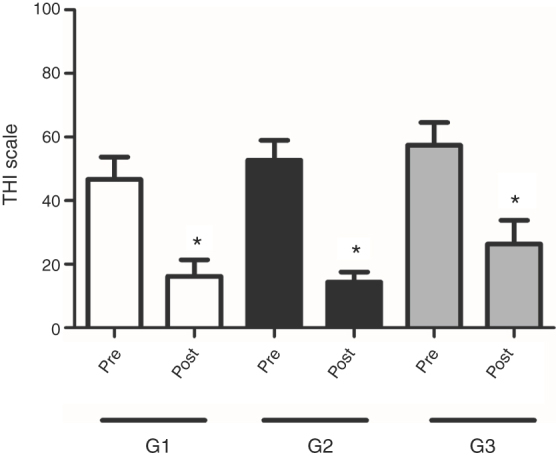
Analysis of the improvement of tinnitus annoyance after the interventions. Averages of THI scores in G1, G2 and G3 groups before and after treatment. Statistical significance (ANOVA repeated measures); *p < 0.05.

**Figure 4:**
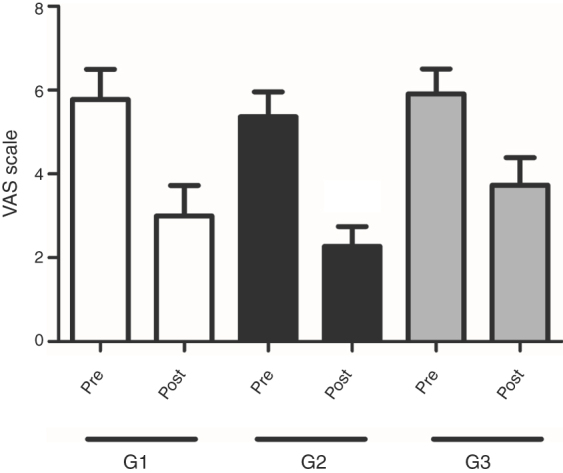
Analysis of improvement of tinnitus discomfort after interventions. Averages of VAS scores in G1, G2 and G3 groups before and after treatment. Statistical significance (ANOVA repeated measures); *p < 0.05.

In the comparison of *G. biloba* extract EGb 761 and/or HA effects on tinnitus, the values were presented as mean and standard deviation of THI and VAS scales. The groups (G1, G2 and G3) were homogeneous in the pre-treatment period, when THI scores (points) were compared within the confidence intervals using the ANOVA test with the application of the Tukey Average Comparison Post Test (*p* = 0.4708) (Fig. 3). The multiple comparisons test also found that there was no significant difference between the groups in the pre-treatment period, when VAS responses regarding the average and standard deviation were considered (*p* = 0.7514) (Fig. 4). Thus, the results of tinnitus evaluation did not differ between the randomized groups in the pre-treatment period (Figure 3, Figure 4).

The results demonstrated an improvement obtained after treatments proposed, taking into consideration THI as the tool applied. It can be observed that in all treatment situations, be it through medication, through the use of hearing aids or both at the same time, there was significant improvement after 90 days of treatment (Fig. 3). This points sharply at the decrease of tinnitus through THI for the three types of treatment proposed (*p* < 0.0001). The results also show that G2 (patients fitted with HA) was also outstanding regarding the improvement of tinnitus when the responses of patients to the THI questionnaire were analyzed (Fig. 3).

In the results obtained after VAS analysis, it was observed that all groups showed a decrease in tinnitus perception and discomfort score. However, this difference was statistically significant only in Group 2 (*p* =  0.0002), when the VAS was applied (Fig. 4).

**Figure 5:**
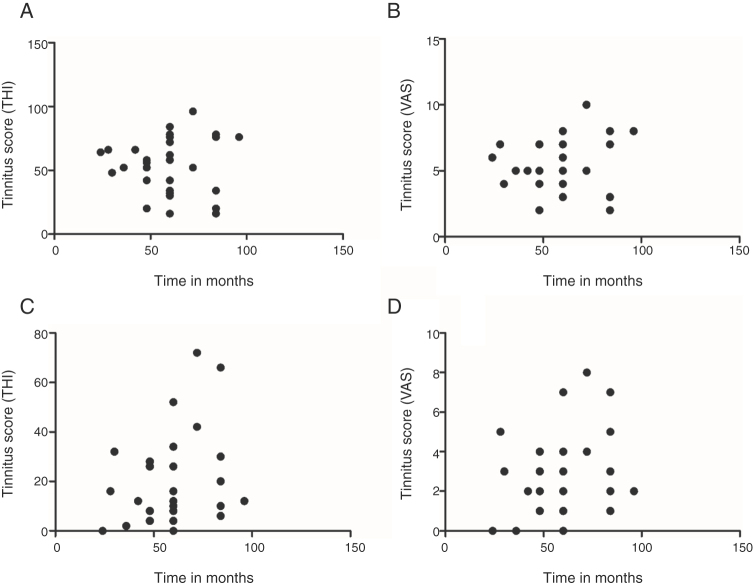
Analysis of the correlation between time and tinnitus score. Representation of THI scores versus time of tinnitus in months, pre-treatment (A) and post-treatment (C). Representation of VAS scores versus time of tinnitus in months, pre-treatment (B) and post-treatment (D).

The correlation between tinnitus onset time and tinnitus perception and discomfort score was evaluated at the different stages of the survey and treatment, with the tinnitus score coming from the THI and VAS. Regarding the evaluation of the correlation between the time of tinnitus and the tinnitus score measured by the THI in the pre-treatment (*r* = 0.0035; *p* =  0.9849) and post-treatment (*r* = 0.2936; *p* =  0.1089) periods, it was found that it was impossible to establish such an association (Fig. 5A and C). In the case of VAS, also in the pre-treatment (*r* = 0.1828; *p* =  0.3250) and post-treatment (*r* = 0.2482; *p* =  0.1782) periods, it was found that there was no significant correlation between tinnitus onset time and the tinnitus THI and VAS scores (Fig. 5B and D).

The mean percentage of variation of the THI score before and after treatment in relation to the tinnitus onset time was also analyzed. The results demonstrated that in the evaluation of tinnitus score, when dividing the groups into subgroups according to tinnitus time, above (Ga) and below (Gb) the mean time of tinnitus from all patients, a discrete correlation between tinnitus onset time and tinnitus perception and discomfort score measured before and after treatment was observed (Table 2). The correlation was better observed in the G2 group (patients fitted with HA), where the variation of the THI score showed improvement of 34.7% in patients with longer tinnitus time (G2a subgroup), while in the subgroup with shorter tinnitus time (G2b) the improvement was 71.2%, much higher than G2a subgroup (Table 2). These results show that the use of the hearing device was more effective the shorter the patients’ tinnitus duration. On the other hand, in groups treated with *G. biloba* extract, G1 and G3, the tinnitus onset time does not seem to affect the treatment effect. The variation of the THI score in the subgroups G1a and G1b was 54.3% and 74.5%, respectively. While in the subgroups G3a and G3b the variation of the THI score was 53.0% and 68.1%, respectively (Table 2). Thus, according to these data, it can be demonstrated that *G. biloba* extract EGb 761 treatment was effective regardless of the patients’ tinnitus duration. In the VAS scale, the difference between the percentage of variation of the scoring score before and after the treatment in relation to the tinnitus time of the patients was not so evident.

**Table 2:** Mean percentage THI score variation before and after treatment in relation to tinnitus time in months. The groups were subdivided into two groups: above (Ga) and below (Gb) the mean tinnitus time of patients in each group.

	Ga	Gb
G1	54.3%	74.5%
G2	34.7%	71.2%
G3	53.0%	68.1%

## Discussion

The heterogeneity of tinnitus is a challenge in clinical tinnitus research as it can differ in many aspects such as localization, sound characteristics, underlying cause, and others. Since there are different forms of tinnitus, one may presume that they differ in pathophysiology and, therefore, in response to specific treatments. This may justify the great variability of the results from clinical studies. Thus an accurate description of the patients under study in a specific trial is mandatory.^23^ Due to the difficulty of measuring and treating the tinnitus symptom, some evaluations required to set up parameters, such as the use of VAS and of questionnaires on the impact on the quality of life of patients and THI.^24^ The present study has used the THI, which is currently the only instrument translated and culturally adapted to Brazilian Portuguese. Although the Tinnitus Functional Index has been developed with the purpose to detect treatment-induced changes and has been recommended as the ideal instrument for outcome measures purpose it was not available in Portuguese at the time of data collection.^25^ Discrepancies are found in the literature regarding the existence of a correlation between the psychoacoustic evaluation of tinnitus and the subjective questionnaires (such as the THI). These questionnaires examine several aspects related with tinnitus (emotional, functional and catastrophic). As for the VAS, it evaluates tinnitus perception and discomfort, which generates a score without specifying which aspect causes the greater discomfort.^26^ Regarding their simplicity, the methods of evaluating the perception, discomfort and severity of tinnitus most used in the published literature are the THI questionnaire and the VAS scale.^21^, ^22^ This study has shown the existence of a significant correlation between the THI and VAS scales (Fig. 2). The substantial correlation found in this study between the THI and VAS tools applied before and after treatment corroborates the data obtained by other authors,^27^ which demonstrates that the use of these two methods would increase the credibility of the surveys.

*G. biloba* is described as an herbal medicine whose active pharmacological groups are flavonoids with antioxidant and vasodilator action, and terpene lactones, which act as antiplatelet agents.^28^ The standardized extract of *G. biloba* leaf EGb 761 results among the main pharmacological activities an increase in cognition, improvement in metabolism and blood flow.^29^ In the present study, treatment with *G. biloba* (Equitam®/EGb 761) has shown to be effective in the reduction and improvement of the tinnitus handicap. The positive result agrees with the beneficial effect found in other clinical studies,^13^, ^30^ and in others diseases such as in dementia.^31^ The most important clinical applications of *G. biloba* extract EGb 761 are in the treatment of tinnitus, acute cochlear deafness, vertigo, and disturbances in equilibrium.^13^, ^14^ Studies with animal models have shown that the treatment with EGb 761 resulted in a decrease of tinnitus when behavioral manifestations were evaluated in mice.^32^ Krauss et al.^33^ demonstrated that the prophylactic application of *G. biloba* extract EGb 761 significantly reduces noise induced hearing loss and tinnitus development in Mongolian gerbil (*Meriones unguiculatus*). *G. biloba* extract also significantly minimizes cochlear damage against LPS-induced otitis media with labyrinthitis in a guinea pig model^34^ and exerts an anti-neuroinflammatory effects in LPS-activated primary microglial cells suggesting a role on neurodegenerative conditions.^35^ EGb 761 is a standardized extract of a well-defined complex mixture of active compounds of heterogeneous chemical composition that give to this phytomedicine the advantage of modulating several factors with mechanism of action such as antioxidant effect, modulation of neurotransmission, neuroendocrine regulation and upregulation of neurotrophic factors.^29^, ^31^ Those are possible mechanisms of action that underline the potential therapeutic effect of EGb 761 on tinnitus, probably regulating the neuromodulation in the central auditory pathway.^36^

On the other hand, the literature also records inconclusive studies regarding the association between *G. biloba* and tinnitus,^30^ and other studies have not found differences after treatment with *G. biloba* in comparison with placebo.^37^, ^38^, ^39^ The conflicting therapeutic results of *G. biloba* as a treatment for tinnitus can be attributed to the lack of standardization of the extracts used, no standard rigorous methodologies regarding the evaluation of therapeutic effectiveness, no optimal dosages and pharmaceutical forms.^15^

In the present study significant improvement was found in the self-perception of tinnitus loudness and severity 90 days after HA fitting. Several forms of intervention are used to minimize tinnitus severity, with HA being mentioned as a therapeutical suggestion.^7^ In patients with tinnitus, sound therapy done with the use of HA aims at auditory stimulation through sound enrichment.^4^ Other studies have also demonstrated the efficiency of the use of HA in the reduction or elimination of tinnitus in patients with hearing loss associated with tinnitus, stating that it is possible to decrease tinnitus perception and severity by enriching the sound environmental (for example, using the HA).^40^, ^41^ This fact can be partially explained by the peripheral auditory masking caused by the amplification of environment sounds, but for many patients this effect is not instantaneous and may take time, which shows the need of taking into account the plastic changes in the central nervous system. Data from the existing literature regarding the adaptation period needed to perceive improvement of tinnitus demonstrated that the time of use of HA needed for this improvement varies from three to eight months for the majority of the subjects studied.

We observe that the associations of *G. biloba* extract EGb 761 with HA showed significant improvement in the pre-treatment and post-treatment comparison, but was not outstanding regarding the improvement of tinnitus in comparison with the group that used either HA or *G. biloba* extract EGb 761 alone. As for the benefits that the medication mentioned offers in clinical practice, as well as in accordance with some of the published studies about the use of hearing aid, it was initially thought that this association had a greater effectiveness than the isolated treatments, but, unexpectedly, we observed the same effect of all treatments. These results demonstrated that was not possible to determine a synergism between the treatments.

No correlation was found between the tinnitus onset time and the tinnitus THI and VAS scores. However, comparing patient's subgroups classified in relation of tinnitus onset time, it was observed that the use of the device was more effective in patients with shorter tinnitus duration. This may be associated with the fact that the patient with a shorter time of tinnitus may not have undergone plastic changes in the central nervous system, so it is often easier to obtain a better clinical response. As time goes by, the loss of compensation mechanism occurs and, consequently, the treatment becomes complex, due to these central nervous system alterations.^42^, ^43^ Also, improved auditory performance and consequently improved tinnitus as well as enhanced communication may reduce the symptoms often associated with tinnitus such as stress or anxiety.^7^ On the other hand, the results demonstrated that the use of *G. biloba* extract EGb 761 alone or in combination with HA, was effective regardless of patient's time tinnitus. This result suggests that improvement of symptoms, regardless of tinnitus onset time, may be associated with *G. biloba's* free radical scavenger activity, anti-inflammatory activity and enhanced neuronal plasticity that can reduce tissue and neurological damage.^29^

It is important to consider the difficulties in the treatment of tinnitus and the lack of treatment options with proven effectiveness. However, the efficacy of cognitive behavioral therapy is well established, and studies have shown a beneficial effect on tinnitus distress. Other methods include medical treatments and psychological approaches in combination with therapeutic sound, such as tinnitus retraining therapy.^3^, ^44^, ^45^ Altogether the results indicate that the treatment options, as used in this study, can achieve satisfactory results and possibly have significant consequences for the care and quality of life in patients with tinnitus. However, there is a need for studies done with a larger number of patients and a longer clinical evaluation time.

The absence of follow-up may be a limitation of this study. Therefore, it was not possible to assess whether treatment with hearing aids and/or *G. biloba* extract EGb 761 had a long-term effect on the perception, intensity, and quality of life in tinnitus individuals. Another limitation was the possibility of the placebo effect on tinnitus intervention, since the control group was not subjected to intervention to rule out this possibility and therefore we cannot know if the effect of placebo treatment would be related to tinnitus improvement. Both the numbers of participants per treatment and the sample size of patients with tinnitus can be seen as a limitation of the research, due to the heterogeneity of triggers and tinnitus mechanisms. So, a larger number of patients and a sample size would be recommended.

## Conclusion

In conclusion, our study demonstrated the effectiveness of treatment with the individual hearing aids and/or *G. biloba* extract EGb 761 associated, through the significant improvement in self-perception of tinnitus loudness and severity, by analysis of the THI score. In addition, hearing aids were more effective in patients with shorter time to onset of tinnitus. *G. biloba* extract alone or in combination with the hearing aids was effective regardless of tinnitus duration. Further studies with high methodological quality and low risk of bias are necessary to evaluate the effect of hearing aids and/or *G. biloba* extract EGb 761 in the treatment of tinnitus. Consort rules should be followed and described in detail, as well as participants’ tinnitus variability.

## Conflicts of interest

The authors declare no conflicts of interest.
